# Significance of radiologic extranodal extension in locoregionally advanced nasopharyngeal carcinoma with lymph node metastasis: a comprehensive nomogram

**DOI:** 10.1016/j.bjorl.2023.101363

**Published:** 2023-11-20

**Authors:** Jianming Ding, Jiawei Chen, Yuhao Lin, Jiabiao Hong, Chaoxiong Huang, Zhaodong Fei, Chuanben Chen

**Affiliations:** Clinical Oncology School of Fujian Medical University, Fujian Cancer Hospital, Department of Radiation Oncology, Fujian, PR China

**Keywords:** Nasopharyngeal carcinoma, Nomogram, Prognosis, Primary gross tumor volume and radiologic extranodal extension

## Abstract

**Objective:**

We aimed to assess the significance of rENE and creat a predictive tool (nomogram) for estimating Overall Survival (OS) in locoregionally advanced Nasopharyngeal Carcinoma (NPC) patients with Lymph Node Metastasis (LNM) based on their clinical characteristics and Radiologic Extranodal Extension (rENE).

**Methods:**

Five hundred and sixty-nine NPC patients with LNM were randomly divided into training and validation groups. Significant factors were identified using univariate and multivariate analyses in the training cohort. Then, the nomogram based on the screening results was established to predict the Overall Survival (OS). Calibration curves and the Concordance index (C-index) gauged predictive accuracy and discrimination. Receiver Operating Characteristic (ROC) analysis assessed risk stratification, and clinical utility was measured using Decision Curve Analysis (DCA). The nomogram's performance was validated for discrimination and calibration in an independent validation cohort.

**Results:**

A total of 360 (63.2%) patients were present with radiologic extranodal extension at initial diagnosis. Patients with rENE had significantly lower OS than other patients. Multivariate analysis identified the five factors, including rENE, for the nomogram model. The C-index was 0.75 (0.71–0.78) in the training cohort and 0.76 (0.69–0.83) in the validation cohort. Notably, the nomogram outperformed the 8th TNM staging system, as evident from the higher AUC values (0.77 vs. 0.60 for 2 year and 0.75 vs. 0.65 for 3 year) and well-calibrated calibration curves. Decision curve analysis indicated improved Net Benefit (NB) with the nomogram for predicting OS. The log-rank test confirmed significant survival distinctions between risk groups in both training and validation cohorts.

**Conclusions:**

We demonstrated the prognostic value of rENE in nasopharyngeal carcinoma and developed a nomogram based on rENE and other factors to provide individual prediction of OS for locoregionally advanced nasopharyngeal carcinoma with lymph node metastasis.

**Level of evidence:**

III.

## Highlights

• Large-case study showed poor survival in cancer with radiologic extranodal extension.
• New predictor for nasopharyngeal cancer survival via radiologic extranodal extension.
• Predictive tool outperformed 8th TNM staging in accuracy and discriminative ability.

## Introduction

Nasopharyngeal Carcinoma (NPC) is the most common malignant tumor in the head and neck region and occurs with high frequency in Southeast Asia and Southern China.^1^ Due to deep location and nonspecific symptoms of the disease, Locoregionally Advanced (LA) diseases in more than 70% of patients. Nodal metastases are highly common among NPC patients with 49%‒85% having them at presentation. The most frequently involved regions include retropharyngeal nodes and level II nodes.^2^ Radical radiation with or without chemotherapy is an effective treatment for NPC. However, the failure of this cancer is primarily due to local recurrence and metastasis, particularly in locoregionally advanced stages with Lymph Node Metastasis (LNM).^3^ Identifying a reliable prognostic system before treatment has important guiding value in formulating the clinical treatment plan and predicting the prognosis. At present, the most widely used system is the American Joint Committee on Cancer TNM staging system (AJCC/TNM). The TNM staging system can distinguish treatment prognosis between patients with early and advanced stage well. However, it appears to reach limitations when discriminating outcomes for patients in the advanced stage.

Numerous studies have underscored the importance of various factors, including Radiologic Extranodal Extension (rENE), Primary gross Tumor Volume (PTV), hematological biomarkers and Standardized Uptake Values (SUV) derived from 18F-FDG positron emission tomography. These factors, although not currently integrated into the AJCC/TNM staging system, served as valuable prognostic indicators for predicting unfavorable outcomes in patients with NPC.^4^, ^5^, ^6^, ^7^, ^8^, ^9^ Notably, rENE has emerged as a particularly significant contributor to prognosis. Through combining the TNM staging system with rENE considerably boosts the accuracy of N staging prediction in the previous studies.^10^, ^11^, ^12^, ^13^ Mao et al. and Lu et al. proposed two new clinical-N classifications base on rENE, leading to more refined survival distinctions across different cN categories.^10^, ^14^ And we can see that the 8th edition of AJCC staging system for head neck has included rENE in the N-stage.^15^ However, the TNM staging system for nasopharyngeal carcinoma remains devoid of rENE inclusion. Consequently, this study delved into the pivotal role of rENE and developed a predictive nomogram based on the findings of preliminary screenings. This nomogram seeked to predict the Overall Survival (OS) of individuals afflicted with LA-NPC accompanied by LNM.

## Methods

### Patients

This retrospective investigation encompassed 569 individuals with newly diagnosed NPC who underwent complete treatment at our cancer center within the timeframe of January 2012 to December 2018. Rigorous participant selection criteria were adhered to, including: a) Biopsy-proven primary NPC; b) Availability of pertinent clinical and laboratory baseline data; c) Pre-treatment whole-body 18F-FDG PET/CT scans and head and neck MRI; d) Stage III‒IVA with lymph node metastasis; e) Radical treatment; To ensure study integrity, certain exclusion criteria were employed, involving: a) The presence of other malignancies; b) Other concomitant fatal disease; c) A history of malignancies treatment. The staging of all participants was meticulously carried out according to the 8th edition of the AJCC/TNM staging system. The study was approved by the Ethical Committee of Fujian Cancer Hospital (YKT2020-011-01).

### MRI and PET-CT examination

Head and neck MRI examinations were performed using either a 1.5 T MR system (Signa Excite 1.5 T HD Twin Speed, GE Healthcare, Milwaukee, Wisconsin) or 3.0 T MR (Achieva 3.0 T, Philips Healthcare, Best, the Netherlands).^16^

For PET/CT imaging, the Gemini TF 64 PET/CT scanner from Philips (the Netherlands) was employed. The 18F-FDG radiochemical purity exceeded 95%, as ensured by the HM-10 cyclotron. The 18F-FDG SUV was determined by calculating the decay corrected tissue activity (nCi/mL), divided by the injected dose of FDG (nCi) and the patient’s body weight (g). We defined SUVmax-T and SUVmax-N as the maximum SUV of the primary tumor and the regional lymph nodes, respectively.

### rENE and other image parameters

The rENE was defined based on the following criteria: a) Lymph node with only indistinct nodal margins or irregular enhanced nodal capsules; b) Infiltration into adjacent adipose tissue and surrounding structures; c) Matted node.^15^ Matted nodes were characterized as clusters of two or more nodes adjacent to each other without intervening fat planes.

The criteria for diagnosing Cervical Node Necrosis (CNN) at MRI were existing a focal area of high signal intensity on T2-weighted images or a focal area of low signal intensity on contrast-enhanced T1-weighted images, with or without a surrounding rim of enhancement. The tumor volume was calculated by the treatment planning software (Pinnacle, version 9.2, Philips Radiation Oncology System, Wisconsin), which could automatically reconstruct a 3D image by manually delineating each MR image.

### Haematological biomarkers

Haematological biomarkers were collected from each patient before treatment including neutrophil count, platelets count, lymphocyte count, hemoglobin, Lactic Dehydrogenase (LDH), albumin and EB-DNA copies were collected. The Neutrophil-Lymphocyte Ratio (NLR) was determined by dividing the absolute neutrophil count by the absolute lymphocyte count. Subsequently, the Systemic Immune-Inflammation Index (SII) was derived by multiplying the absolute platelet count with the NLR. Finally, the Prognostic Nutrition Index (PNI) was computed by multiplying the absolute lymphocyte count by the sum of the serum albumin level +5.

### Treatment

Platinum-based chemotherapy was administered to a predominant majority of the patient cohort, encompassing 561 individuals (98.6% out of 569). Among the entire group, 551 cases (96.8%) underwent neoadjuvant chemotherapy, 419 cases (73.6%) received concurrent chemotherapy, and 132 cases (23.2%) received adjuvant chemotherapy. The most frequently employed regimen involved the combination of platinum with paclitaxel or gemcitabine. Intensity-Modulated Radiotherapy (IMRT) was applied for radiotherapy. The target volume and radiotherapy dose were implemented according to a previously described protocol.^17^ The radiotherapy prescription dose was GTV 70∼72.6 Gy/31∼33 F, CTV1 62∼62.7 Gy/31∼33 F, and CTV2 54.4∼56.2 Gy/31∼33 F.

### Cutoff values of continuous variables

Continuous variables were transformed into categorical variables. Cut-off values were calculated using Receiver Operating Characteristic (ROC) curve and were as follows: age (53 years), SUVmax-T (7.86), SUVmax-N (10.28), PTV (42.75 mL), NTV (34.88 mL), ALB (32 g/L), LDH (210 U/L), PLR (85.43), NLR (2.60), PNI (59.6), SII (633.9), EB-DNA (18,700).

### Follow-up and endpoints

Patients were followed by routine examination every 3-months for 2-years, every 6-months for years 3–5, then annually. The primary endpoint of this study was the assessment of Overall Survival (OS). This outcome measure was defined as the duration between the initial diagnosis date and either the date of death due to any cause or the date of the most recent follow-up.

### Statistical analysis

Statistical analyses were performed using IBM SPSS Statistical software version 19.0 (IBM Corp., Chicago, IL, USA) and R version 3.4.0 (http://www. R-project.org/).

Patients were randomly divided into a training cohort and validation cohort at a ratio of 7:3 for construction and validation of the nomogram. Variables demonstrating a significant level of statistical significance at *p* <  0.1 in the univariate analyses were subsequently subjected to multivariable Cox regression analysis. Independent prognostic factors were identified if their impact remained statistically significant within the Cox model (*p* <  0.05).

Using these identified prognostic factors, we constructed a nomogram to effectively predict the 2-, and 3-year OS rates. The discriminative power of the nomogram was assessed through the Concordance index (C-index) and the area under the ROC curve analysis. A calibration curve was employed to juxtapose the observed probability against the predicted probability. Furthermore, the clinical validity of the nomogram was demonstrated using Decision Curve Analysis (DCA), which quantified net benefits across varying threshold probabilities. Subsequently, ROC curve was applied to stratify patients into two distinct risk groups. Kaplan-Meier survival analysis was then employed to generate survival curves for these different risk groups, with comparison facilitated by the log-rank test.

## Results

### Patient characteristics and survival

A total of 569 patients were enrolled in this study, with 398 individuals (69.9%) allocated to the training cohort and the remaining 171 (30.1%) to the validation cohort. Baseline characteristics were summarized in Table 1. Importantly, no statistically significant differences were observed in the baseline characteristics between the training and validation cohorts (*p* = 0.114–0.970), except NLR (*p* = 0.042). A total of 360 (63.2%) patients were presence of radiologic extranodal extension at initial diagnosis, including 261 (61.8%) in training cohort and 114 (66.7%) in validation cohort.

**Table 1:** Characteristics of LA-NPC patients with LNM in the training and validation cohorts.

Characteristic	Training cohort	Validation cohort	*p*
*n*	398	171	
Sex, *n* (%)			0.297
Female	284 (71.4%)	130 (76%)	
Male	114 (28.6%)	41 (24%)	
Age (years), *n* (%)			0.502
≤53	271 (68.1%)	122 (71.3%)	
>53	127 (31.9%)	49 (28.7%)	
Tumor stage, *n* (%)			0.905
T1 + 2+ 3	313 (78.6%)	133 (77.8%)	
T4	85 (21.4%)	38 (22.2%)	
Node stage, *n* (%)			0.970
N1 + 2	284 (71.4%)	123 (71.9%)	
N3	114 (28.6%)	48 (28.1%)	
TNM stage, *n* (%)			0.639
III	215 (54%)	88 (51.5%)	
IVA	183 (46%)	83 (48.5%)	
CNN, *n* (%)			0.114
No	235 (59%)	88 (51.5%)	
Yes	163 (41%)	83 (48.5%)	
rENE, *n* (%)			0.314
No	152 (38.2%)	57 (33.3%)	
Yes	246 (61.8%)	114 (66.7%)	
T SUVmax, *n* (%)			0.848
≤7.86	137 (34.4%)	61 (35.7%)	
>7.86	261 (65.6%)	110 (64.3%)	
PTV (mL), n (%)			0.778
≤42.75	345 (86.7%)	146 (85.4%)	
>42.75	53 (13.3%)	25 (14.6%)	
N SUVmax, *n* (%)			0.429
≤10.28	253 (63.6%)	102 (59.6%)	
>10.28	145 (36.4%)	69 (40.4%)	
NTV (mL), *n* (%)			0.203
≤34.88	103 (25.9%)	35 (20.5%)	
>34.88	295 (74.1%)	136 (79.5%)	
ALB (g/L), *n* (%)			0.668
≤32	288 (72.4%)	120 (70.2%)	
>32	110 (27.6%)	51 (29.8%)	
PNI, *n* (%)			0.836
≤59.6	364 (91.5%)	158 (92.4%)	
>59.6	34 (8.5%)	13 (7.6%)	
LDH (U/L), *n* (%)			0.753
≤210	325 (81.7%)	137 (80.1%)	
>210	73 (18.3%)	34 (19.9%)	
SII, *n* (%)			0.850
≤376.4	107 (26.9%)	48 (28.1%)	
>376.4	291 (73.1%)	123 (71.9%)	
NLR, *n* (%)			0.042
≤2.6	277 (69.6%)	134 (78.4%)	
>2.6	121 (30.4%)	37 (21.6%)	
EB DNA (copy/mL), *n* (%)			0.788
≤18,700	290 (72.9%)	122 (71.3%)	
>18,700	108 (27.1%)	49 (28.7%)	

CNN, Cervical Node Necrosis; rENE, Radiologic Extranodal Envasion; PTV, Primary gross Tumor Volume; NTV, Node Tumor Volume; ALB, Albumin; PNI, Prognostic Nutrition Index; LDH, Lactic Dehydrogenase; SII, Systemic Immune-Inflammation index; NLR, Neutrophil-Lymphocyte Ratio.

**Figure 1:**
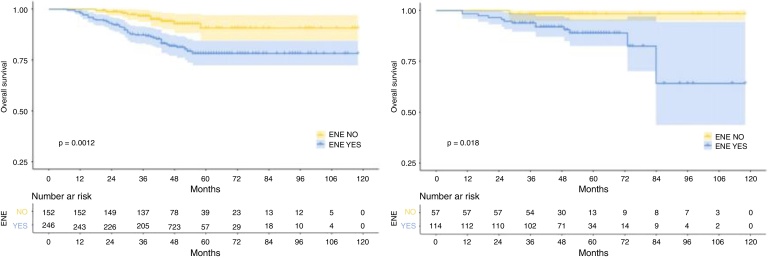
Kaplan-Meier survival curves for rENE of the training cohort (A) and validation cohort (B).

The median follow-up duration for the training cohort was 48 months (range = 7 to 118 months), while the validation cohort had a median follow-up of 49 months (range = 10 to 118 months). Over the course of follow-up, a total of 55 patients in the training cohort and 14 patients in the validation cohort had reached the endpoint of mortality. In addition, the 1-, 3-, and 5-year OS were 98.7%, 90.8%, 82.9%, and 98.8%, 95.3%, 91.8% for the training and validation cohorts, respectively. As expected, patients with rENE had significantly poor prognosis than other patients in both training and validation cohorts (*p* =  0.0012, *p* =  0.018, respectively) (Fig. 1).

### Features selection

Univariate analysis indicated that rENE (*p* < 0.002), age (*p* <  0.012), Tumor stage (*p* =  0.014), Node stage (*p* <  0.008), SUVmax-T (*p* =  0.043), SUVmax-N (*p* =  0.011), PTV (*p* <  0.001), LDH (*p* <  0.001) and EBV DNA (*p* <  0.021) were associated with OS of NPC patients. In multivariate analysis for OS with Cox regression, the results showed that rENE (*p* <  0.002), age (*p* <  0.012), PTV (*p* <  0.001), Node stage (*p* <  0.008) and LDH (*p* <  0.001) remained independently prognostic. The detailed results of univariate and multivariate analyses are presented in Table 2.

**Table 2:** Univariate and multivariate analyses of risk factors in LA-NPC patients with LNM.

Characteristics	Univariate analysis		Multivariate analysis	
	Hazard ratio (95% CI)	p-value	Hazard ratio (95% CI)	p-value
Sex				
Female/male	0.770 (0.413–1.434)	0.409		
Age				
≤53/>53	1.972 (1.161–3.349)	0.012	2.163 (1.262–3.704)	0.005
Tumor stage				
T1 + 2+ 3/T4	2.004 (1.149–3.494)	0.014	1.100 (0.480–2.519)	0.821
Node stage				
N1 + 2/N3	2.061 (1.210–3.512)	0.008	2.046 (1.090–3.840)	0.026
CNN				
No/Yes	1.248 (0.735–2.119)	0.413		
rENE				
No/Yes	2.935 (1.479–5.823)	0.002	2.384 (1.138–4.993)	0.021
T‒SUVmax				
≤7.86/>7.86	1.934 (1.020–3.669)	0.043	1.544 (0.757–3.149)	0.233
PTV				
≤42.75/>42.75	3.026 (1.690–5.417)	<0.001	3.448 (1.356–8.768)	0.009
N‒SUVmax				
≤10.28/>10.28	1.979 (1.166–3.359)	0.011	1.119 (0.595–2.105)	0.728
NTV				
≤34.88/>34.88	1.584 (0.798–3.142)	0.189		
ALB				
≤32/>32	0.889 (0.476–1.661)	0.713		
PNI				
≤59.6/>59.6	1.249 (0.497–3.140)	0.636		
LDH				
≤210/>210	3.482 (2.016–6.013)	<0.001	2.394 (1.304–4.394)	0.005
SII				
≤376.4/>3 76.4	1.162 (0.624–2.165)	0.636		
NLR				
≤2.6/>2.6	0.986 (0.557–1.748)	0.962		
EB‒DNA				
≤18,700/>18,700	1.901 (1.102–3.281)	0.021	0.742 (0.386–1.426)	0.370

CI, Confidence Interval; HR, Hazard Ratio; CNN, Cervical Node Necrosis; rENE, Radiologic Extranodal Extension; PTV, Primary gross Tumor Volume; NTV, Node Tumor Volume; ALB, Albumin; PNI, Prognostic Nutrition Index; LDH, Actic Dehydrogenase; SII, Systemic Immune‒Inflammation Index; NLR, Neutrophil‒Lymphocyte ratio.

### Nomogram construction

**Figure 2:**
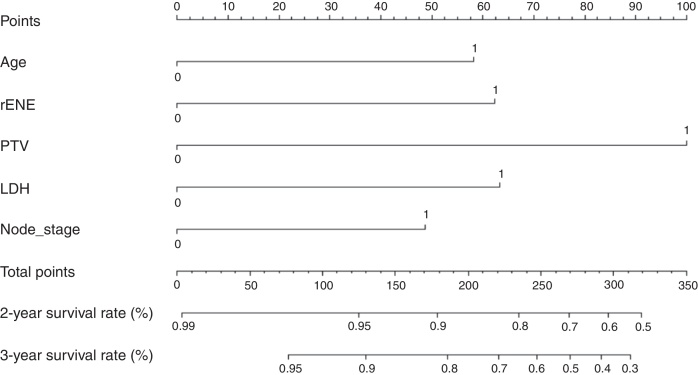
Nomogram model predicting 2 and 3 year OS in LA-NPC patients with LNM.

**Figure 3:**
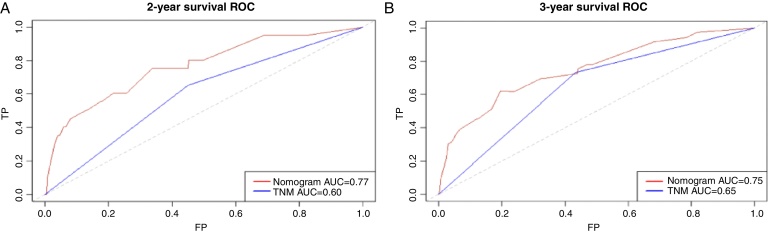
Receiver operating characteristic (ROC) curves for nomogram (A) and TNM staging system (B) for 2-year and 3-year overall survival rate in the training cohort.

**Figure 4:**
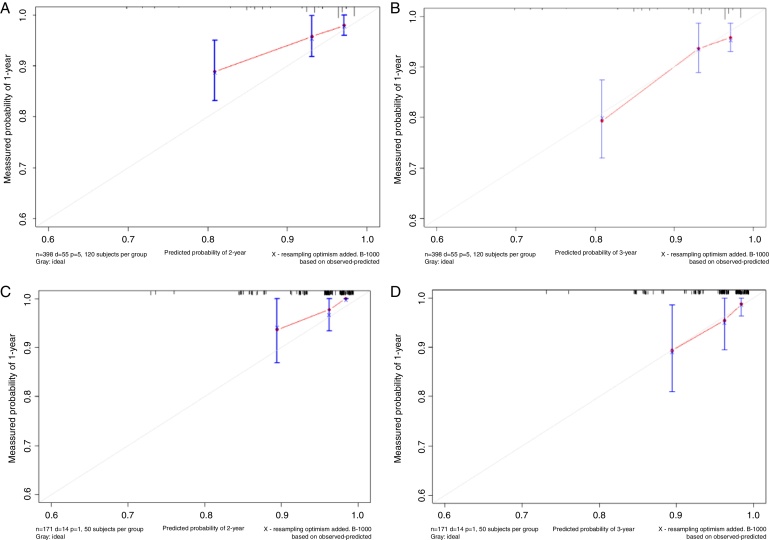
Calibration curves of the nomogram predicted and actual measured survival probabilities at 2- and 3-years of training cohort (A and B) and validation cohort (C and D).

**Figure 5:**
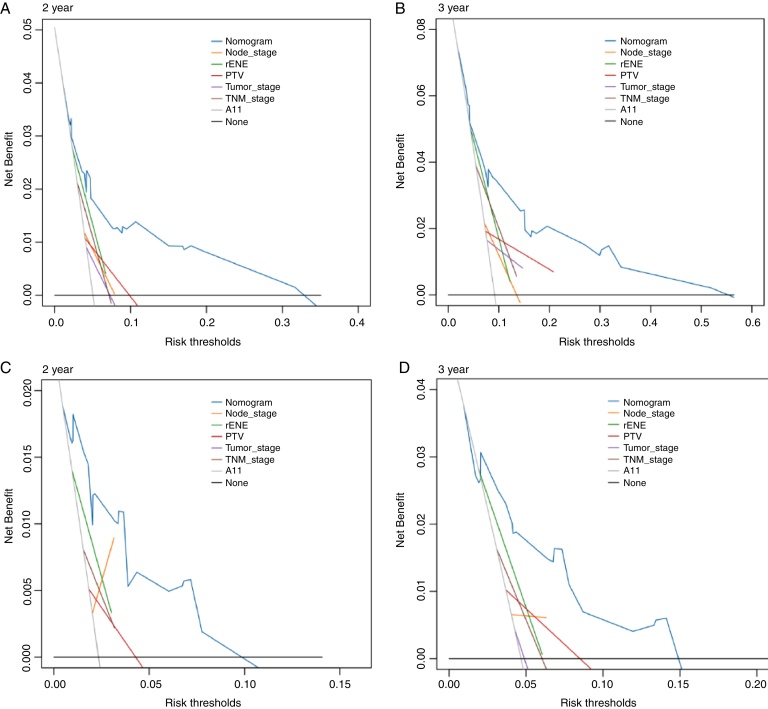
Decision curve analysis for 2-year and 3-year survival predictions of training cohort (A and B) and validation cohort (C and D).

Based on the above significant independent factors selected from the training cohort, a nomogram was constructed to predict OS for LA-NPC with LNM at 2-, and 3-years (Fig. 2), where larger points indicated a shorter OS. The C-index of the nomogram for OS was 0.75 (0.710–0.78). The AUCs of the nomogram (0.77 for 2-year; 0.75 for 3-year) were significantly better than the 8th edition of the TNM staging system (0.60 for 2-year; 0.65 for 3-year) in predicting 2- and 3-year OS (Fig. 3A and B). The calibration plots revealed an excellent prediction of 3-year OS and an acceptable prediction of 2-year OS (Fig. 4A and B). Ultimately, the DCA conducted within the training cohort demonstrated a consistently favorable net benefit associated with the nomogram among most threshold probabilities. This suggested a promising clinical utility for the nomogram model. (Fig. 5A and B).

### Nomogram validation

The nomogram was validated in the validation cohort. The C-index of the nomogram in validation cohort for predicting OS was 0.76 (0.69–0.83). The calibration curve underscored the well-calibrated nature of the nomogram's predictions. Notably, the robust concordance observed between actual outcomes and nomogram predictions was particularly pronounced for the 3-year OS, as illustrated in Fig. 4 C and D. The DCA outcomes within validation cohort closely mirrored those observed in the training cohort (Fig. 5C and D).

### Risk stratification

**Figure 6:**
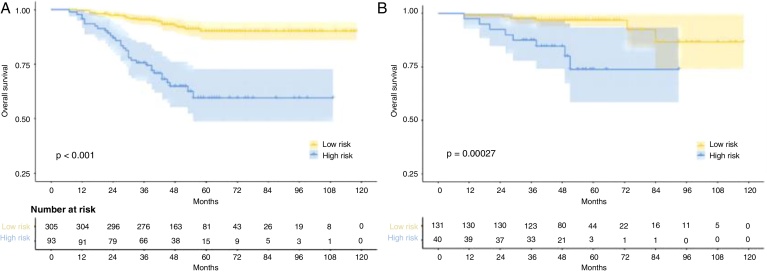
Kaplan-Meier survival curves for different risk groups of the training cohort (A) and validation cohort (B).

The nomogram scores were calculated according to the total scores of each covariate in the model. The cutoff value of the nomogram scores for the stratification of OS was 162.4 by ROC curve. Then the cohorts were divided into high-risk and low-risk groups. The log-rank test disclosed a significant divergence in survival outcomes, a finding that held true within both the training cohort (*p* <  0.001) (Fig. 6A) and the validation cohort (Fig. 6B). Therefore, the risk stratifications could effectively discriminate OS for the proposed risk groups in LA-NPC with LNM.

## Discussion

In our retrospective study, we successfully found the prognosis value of rENE and developed a nomogram model for predicting OS in LA-NPC patients with LNM. Patients with rENE had significantly lower OS than other patients. Drawing upon multivariable analyses, we integrated five independent prognostic factors into the predictive nomogram model, including rENE, PTV, LDH, N-stage and age. Our model's enhanced predictive accuracy and discriminative capacity surpassed that of the widely employed TNM staging system. The model enabled effective stratification into high-risk and low-risk groups.

The diagnosis of rENE entails identification of indistinct nodal margins, irregular nodal capsular enhancement, matted nodes, or infiltration into adjacent fat or structures on MR imaging.^18^ Numerous studies have demonstrated the prognostic value of rENE and its underlying mechanism. The muscular fascia is a strong protective barrier against tumor infiltration and the structure was surrounded by branches of vessels, which provide blood supply. When the fascia is ruptured, the entire muscle is put in danger because cancer cells can spread rapidly through the fatty tissue that runs along the muscle fibers when the fascia is damaged.^19^ Still, tumor cells can invade the blood circulatory system through vessels, causing treatment failure.^20^, ^21^ Owing to substantial evidence on its potential effect on survival, the 8th edition of AJCC staging system for head neck has included rENE in the N-stage.^15^ However, the role of rENE in nasopharyngeal carcinoma remains controversial. Ai et al. showed that rENE based on MRI was an independent prognostic factor.^21^ However, Guo and Li reported negative results.^22^, ^23^ The present study revealed that rENE was associated with worse OS. This contradictory result may be due to the subjective diagnosis of rENE without significant invasion of adjacent tissues, which varies greatly between radiologists. For example, the fat infiltration around the tumor was one of the strongest imaging characteristics that helped confirm the clinical diagnosis of rENE in King et al. and Lodder et al.’s studies.^24^, ^25^ But in Hu et al.’s study, fat infiltration was an unstable imaging sign that the results could not be easily duplicated, with relatively low inter-rater and intra-rater concordance.^26^ Hence, much work remains to be done to incorporate rENE into staging system, including precise defining and grading of the rENE.

At present, the management of NPC in advanced stage is particularly challenging due to poor prognosis. After our literature review, there are few predictive models for this subgroup. Jiang et al. developed a nomogram for OS based on the variables of age, N-stage, LDH, NLR and T-stage.^27^ Zhao et al. also established a nomogram for OS including the variables of age, N-stage, gender, EB-DNA, T-stage, NLR, LMR, LAR and PNI.^28^ Both models have variables of age and N-stage in common, which were further confirmed that age and N-stage were reliable indicators for prognosis. Many studies have reported that tumor volume is an independent prognostic factor.^4^, ^29^, ^30^ Chen et al. reported that it was an independent prognostic factor in overall survival and suggested it should be included in the TNM staging system.^4^ In our study, the multivariate analysis results confirmed that PTV influenced overall survival. Decision curve analysis revealed a superior NB associated with PTV compared to T-stage in predicting OS within both the training and validation cohorts. This substantiated the notion that incorporating PTV into the T-stage could notably enhance the prognostic capacity, underscoring its potential value in refining the T-stage assessment.^30^, ^31^, ^32^, ^33^ Hematological biomarkers have been extensively reported to influence prognosis recently, such as EB-DNA, LDH, SII, NTR, ALB and PNI.^5^, ^28^, ^34^, ^35^, ^36^ Among these variables, the LDH and EB-DNA had worse impact on survival in the univariate analysis and only the LDH remained significant after multivariate analysis in this study. Numerous studies have investigated the correlation between elevated serum LDH level at diagnosis and survival outcomes in NPC.^34^ Nevertheless, the results were inconsistent and controversial. Our findings distinctly established serum LDH as an independent and predictive factor. Notably, certain scholars had posited a hypothesis suggesting that serum LDH levels could potentially mirror the degree of tumor hypoxia. This premise stems from LDH's role in catalyzing the conversion of pyruvate to lactate under hypoxic conditions.^37^ Tumor cells are resistant to chemoradiotherapy in the hypoxia conditions, which in turn leads to treatment failure. Increasing research had confirmed that plasma EBV DNA was an important biomarker for prognostication and tumor monitoring. This was confirmed by our univariate analysis results but was not confirmed by multiple analysis results. It may be attributable to a certain collinearity with other variables which will be automatically eliminated in multivariate analyses.

Our study has several limitations. First, this is a retrospective study and thus subject to potential selection biases. Therefore, the prospective studies need to be carried out to confirm the results. Second, the study is from one institution with a small sample size, our results should be verified by more samples and other institutions. Third, to achieve a more accurate outcome, rENE should be well graded. More information was needed in the following study to identify different grades of rENE’s impact on the prognosis. Also, the discrepancy between different radiologists and different institutions limited the clinical use of rENE and should be noticed. Finally, our study is limited to LA-NPC patients with LNM and has no guiding significance for patients in other stage.

## Conclusion

In summary, we demonstrated the prognostic value of rENE in nasopharyngeal carcinoma and developed a nomogram based on rENE and other factors to provide individual prediction of OS for locoregionally advanced nasopharyngeal carcinoma with lymph node metastasis. Our model showed higher predictive accuracy and independent discriminative ability compared with the 8th TNM staging system. The rENE could be incorporated in the N stage. Further large-scale and multi-center studies with well-designed methods are warranted to confirm the present results in the future.

## Consent for publication

Not required.

## Availability of data and material

Data are available upon reasonable request.

The data sets generated during and/or analyzed during the current study are available from the corresponding author on reasonable request.

## Authors’ contributions

Study concept and design: CB C, ZD F. Acquisition, analysis, or interpretation of data: All authors. Drafting of the manuscript: JM D, JW C, YH L. Critical revision of the manuscript for important intellectual content: All authors. Statistical analysis: JB H, CX H. Study supervision: CB C.

## Funding

This work was supported by research projects for Bethune-Translational Medicine Research Fund for Oncology radiotherapy (flzh202126).

## Conflicts of interest

The authors declare no conflicts of interest.
